# Innovation in Aircraft Cabin Interior Panels Part I: Technical Assessment on Replacing the Honeycomb with Structural Foams and Evaluation of Optimal Curing of Prepreg Fiberglass

**DOI:** 10.3390/polym13193207

**Published:** 2021-09-22

**Authors:** Edgar Adrián Franco-Urquiza, Annika Dollinger, Mauricio Torres-Arellano, Saúl Piedra, Perla Itzel Alcántara Llanas, Victoria Rentería-Rodríguez, Cecilia Zarate Pérez

**Affiliations:** 1 National Council for Science and Technology (CONACYT—CIDESI), Center for Engineering and Industrial Development, Carretera Estatal 200, km 23, Querétaro 76265, Mexico; saul.piedra@cidesi.edu.mx; 2 Institute of Aviation, Department of Engineering, University of Applied Sciences, FH JOANNEUM GmbH, 8020 Graz, Austria; annika.dollinger@edu.fh-joanneum.at; 3 Center for Engineering and Industrial Development (CIDESI), Carretera Estatal 200, km 23, Querétaro 76265, Mexico; mauricio.torres@cidesi.edu.mx (M.T.-A.); perla.alcantara@cidesi.edu.mx (P.I.A.L.); ana.renteria@cidesi.edu.mx (V.R.-R.); cecilia.zarate@cidesi.edu.mx (C.Z.P.)

**Keywords:** aircraft cabin interior panels, optimal curing of prepregs, foams, non-structural composite panels

## Abstract

Sandwich composites are widely used in the manufacture of aircraft cabin interior panels for commercial aircraft, mainly due to the light weight of the composites and their high strength-to-weight ratio. Panels are used for floors, ceilings, kitchen walls, cabinets, seats, and cabin dividers. The honeycomb core of the panels is a very light structure that provides high rigidity, which is considerably increased with fiberglass face sheets. The panels are manufactured using the compression molding process, where the honeycomb core is crushed up to the desired thickness. The crushed core breaks fiberglass face sheets and causes other damage, so the panel must be reworked. Some damage is associated with excessive build-up of resin in localized areas, incomplete curing of the pre-impregnated fiberglass during the manufacturing process, and excessive temperature or residence time during the compression molding. This work evaluates the feasibility of using rigid polyurethane foams as a substitute for the honeycomb core. The thermal and viscoelastic behavior of the cured prepreg fiberglass under different manufacturing conditions is studied. The first part of this work presents the influence of the manufacturing parameters and the feasibility of using rigid foams in manufacturing flat panels oriented to non-structural applications. The conclusion of the article describes the focus of future research.

## 1. Introduction

The composite materials market is continually growing in the aerospace sector. Other industries such as automobiles, trains, and components such as tanks or pressure vessels, require these materials with excellent mechanical properties [1]. The extraordinary characteristics of composite materials, also known as fiber-reinforced polymer (FRP) composites, provide new approaches in designing and fabricating complex structures [2,3,4,5]. These composites are already ingrained in current engineering, but research is still in its infancy. According to Market & Markets Global Aircraft Cabin Interiors Market Forecast, airline expenditures account for more than 30% of total fuel operating costs, increasingly demanding more fuel-efficient aircraft [6]. This led to a focus on lighter materials in aircraft, and therefore fiberglass and carbon fiber composites have been used for the past several decades to reduce the weight of panels for cabin interiors. According to the market analysis, composite panels in aircraft interiors could double, from 20–25% currently used to 30–40% [7].

The aeronautical industry is moving today to modernize cabin interiors satisfying three main criteria: commercial viability, certification, and passenger experience. Some of the most notable moves are the development of lightweight and custom cabin solutions to enhance the overall passenger experience; an expansion of the overhead storage compartments to accommodate more luggage per passenger; the design of a maximum cabin space that allows comfort and the feeling of space, but optimizing the space to add more seats. In this sense, composite materials represent an indispensable focus area for the aircraft cabin design without sacrificing performance, safety, and cost [8,9].

Aircraft cabin interiors have witnessed a significant and impactful evolution over the past decades as composite materials have taken a disruptive journey and played a critical role in enhancing cabin interiors in general. The interiors of aircraft cabins play an essential role in improving the comfort of passengers on board. Based on the type of interior, the aircraft cabin interior market has been segmented into aircraft seating, inflight entertainment and connectivity, cabin lighting, galley equipment, aircraft lavatories, aircraft windows and windshields, overhead compartments, and divider panels. However, the materials used in the cabin interiors are similar regardless of airline or type of aircraft since all passenger-carrying craft must meet the flammability requirements [10,11,12].

Aircraft seats are the product most in demand in the aircraft cabin interior market, while galley equipment is the most expensive [8,9,13]. The interiors of aircraft cabins are a combination of different products, and each product has its function. In this sense, the primary function of aircraft seats is to provide comfort and safety to passengers. The requirements of the aircraft cabin interior panels present a good balance between strength, low density, high stiffness, and durability. In addition, most panels require contoured forms, which lead to several defects induced during the manufacturing process [14]. Aircraft cabin interior panels are manufactured using typical sandwich composites configuration made with aramid honeycomb core bonded to outer layers or face sheets, commonly glass fiber epoxy prepreg. A lightweight, low-density core between thin sheets dramatically increases panel rigidity with little added weight. Flat and complex panels are fabricated using single-opening and multi-opening presses, respectively. For molds with complex geometries, the one-step compression molding leads to distinct types of induced failures, such as breaks, holes, and the absence of impregnated zones or areas with excessive resin accumulation in the contours of the mold [15,16,17]. This occurs when the sandwich composite is placed in a large press and crushed to a predetermined shape and thickness. Then, the crushed honeycomb acts like multiple blades that break the glass fiber prepreg during the transformation process.

The use of new alternative composite materials and diverse manufacturing technologies boasts many opportunities to develop composite panels, addressing significant challenges like reducing manufacturing costs, ensuring defect-free and repeatable manufacturing, increasing the efficiency of joining technologies, and developing structural maintenance and repair technologies.

This work evaluates the optimal curing conditions of prepreg fiberglass and the mechanical properties of honeycomb and vinyl foam cores to evaluate their viability in developing sandwich composites. This first part aims to show the thermal and mechanical results for the development of sandwich panels, which are mechanically evaluated, and their results are presented in the second part of this work in a later article.

## 2. Materials and Methods

The prepreg fiberglass DA 4080 from APCM (Adhesive Prepregs for Composite Manufacturers), LLC, was used as the skins of the sandwich composites evaluated in this work. DA 4080 is a one-sided pre-impregnated modified epoxy prepreg with a cure temperature of 200 °F (93 °C). The prepreg reinforcement is an E-glass fabric made up of a 200 gsm layer of bidirectional fabric.

The core used was the PN1 honeycomb, manufactured by DuPont Nomex^®^ and purchased to Plascore. The PN1 is a sheet with regular hexagonal cell configuration, approximately 0.5 inch/13 mm thick, and a 0.12 inch/3 mm cell size.

**Figure 1:**
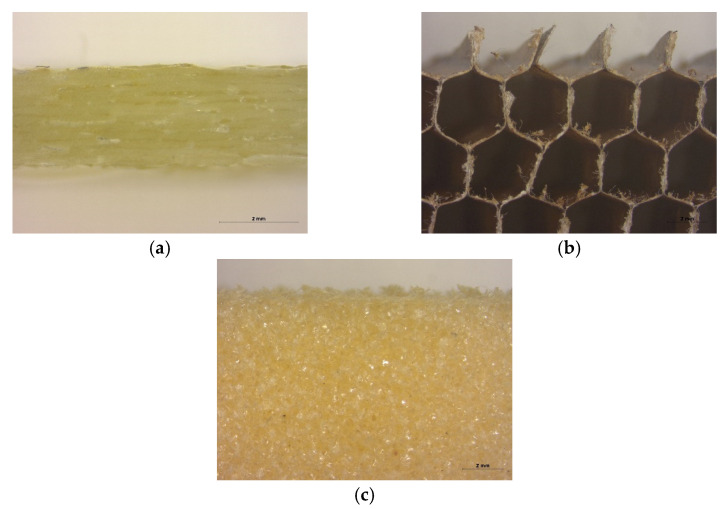
Pictures of the individual materials used to manufacture distinct sandwich composites: (**a**) glass fiber prepreg, (**b**) honeycomb core, (**c**) foam core.

The another core used was the 3 lb. density lightweight DIAB 1022 Vynil foam Divinycell^®^ supplied by Fibreglast. According to the supplier, the DIAB 1022 offers the highest strength to density ratio, insulative properties, improved stiffness, and impact resistance. The 0.5″ (12.7 mm) thick foam is best when only additional impact resistance is required. This foam can be easily thermoformed with a heat gun or oven. Other properties of this foam are excellent adhesion/peel strength, chemical resistance, good dimensional stability, and temperature performance. The operating temperature can reach from −200 °C to 70 °C. The processing temperature depends on time and pressure conditions. Figure 1 shows some pictures of the materials used.

Three different manufacturing methods for sandwich constructions are autoclave, press, or vacuum bag molding. For processing within an autoclave or press, all parts of a sandwich panel are cured in a single process, called co-curing. In vacuum bag molding, more stages of lay-up and curing are necessary. Co-curing means that face sheet composite and the adhesive are cured simultaneously. This work will mainly address the co-curing method with the production technology compression molding.

Fiberglass fabric epoxy prepreg (GF) laminates of two plies with the same configuration (0/90°) were fabricated using a hot plate press CARVER model 4122, at 200 °F (93 °C) with a constant pressure of 20 psi during 1 h, as recommended by the supplier. Additionally, the manufacturing parameters on the fiberglass prepreg DA 4080 (GF) curing process were evaluated. Therefore, distinct combinations of time and temperature were used to fabricate several GF laminates (Table 1) in order to find the optimal curing parameters. For reference, the GF laminate labeled as 0 corresponds to the non-cured GF. A temperature of 200 °F is recommended on the prepreg fiberglass technical datasheet. According to the manufacturer, the temperature of 300 °F is the maximum service temperature of the honeycomb. The 250 °F temperature is half of both initially proposed temperatures. In an attempt to investigate a different temperature range between 200 and 250 °F, it was decided to increase the curing temperature of the prepreg fiberglass by 19 °F, having 219 and 238 °F. In addition, it was determined to evaluate the temperature above and below 250 °F in +/− 6 °F intervals, resulting in 256 and 244 °F

Differential scanning calorimetry (DSC) was used to obtain information about the optimal curing process of GF. Since epoxy reactions release energy during cure due to their exothermic behavior, DSC can monitor their cure process. The curing evaluation was performed in a DSC 250 of TA Instruments. Samples of the GF laminates of approximately 5 mg were placed in an aluminum pan with a sealed lid and placed opposite the empty reference pan in the oven chamber. The DSC method used was first heating-cooling-second heating, from 10 to 200 °C, and the heating rate of 10 °C/min.

**Figure 2:**
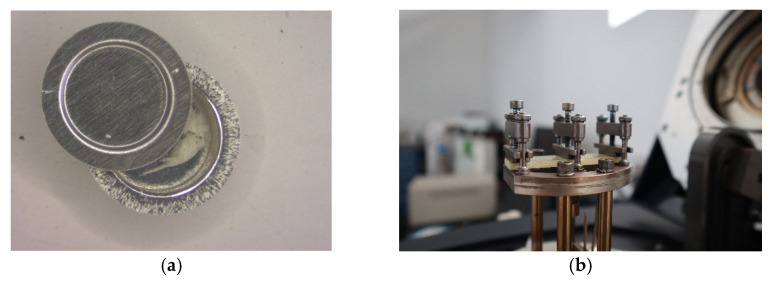
Pictures of representative tests of: (**a**) samples in the aluminum pan before hermetic close and test by differential scanning calorimetry (DSC), (**b**) specimen of glass fiber before testing in dynamic mechanical analysis (DMA).

Dynamic mechanical analysis (DMA) tests were performed to determine the GF laminates’ glass transition temperature (T_g_). Specimens with 50 mm × 12 mm × 3 mm nominal dimensions were tested using the single cantilever geometry to conduct measurements from room temperature to 190 °C with a heating ramp of 5 °C/min in a DMA Discovery 850 from TA Instruments. Storage modulus (E′) and damping factor (tan δ) curves were obtained following the ASTM-D7028. Figure 2 shows the pictures of samples and specimens before tests in DSC and DMA.

Uniaxial compression tests were performed following the ASTM D1621-16 and ASTM C365/C365M-16 for the PN1 honeycomb core (HC) and the rigid cellular polyurethane foam DIAB 1022 (foam), respectively. Square HC and foam (80 × 80 × 12.7 mm) specimens were cut using a universal cutting tool ZUND XL-3200 and tested in a universal testing machine MTS. Insight with a cell load of 30 kN. The tests were performed at room temperature and a crosshead speed of 0.5 mm/min.

**Figure 3:**
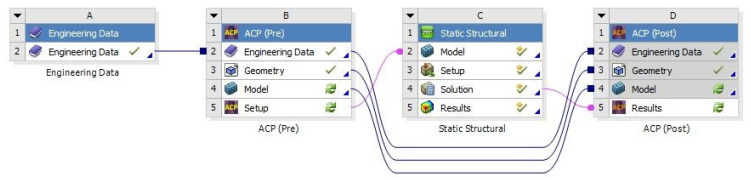
Workflow for the typical ACP usage of a sandwich analysis in ANSYS.

The analysis of sandwich composite panels follows a particular procedure in the software package called ANSYS Composite PrepPost (ACP), the workflow of which can be seen in Figure 3. The first step is to import the geometry and look for any required geometry repair to provide a good mesh before linking it to ACP (Pre). In ACP (Pre) the engineering data are specified, that is to say the material data of the composite like glass fiber prepreg, foam, and honeycomb core. These data can be selected from the Composite Materials Library at Engineering Data Sources. Also, it is possible to generate new material data. In ANSYS Mechanical, the shell mesh of the component is generated, and the last step of ACP (Pre) is to set up composite material fabric, element orientations, or ply lay-up. After this setup, a range of analyses can be solved.

In the static structural analysis loads, boundary conditions and global post-processing of the solution are simulated, and post-processing of composite-specific results like failure criteria and stress or strain fields through the thickness of the component then follows.

## 3. Results

**Figure 4:**
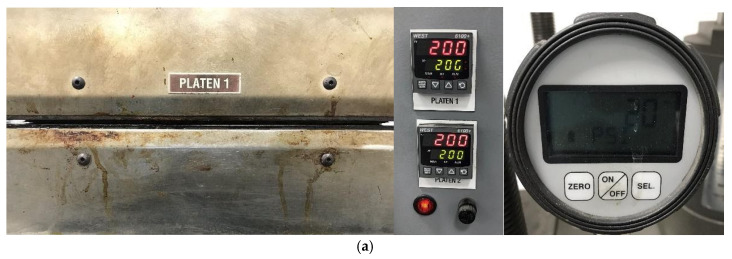

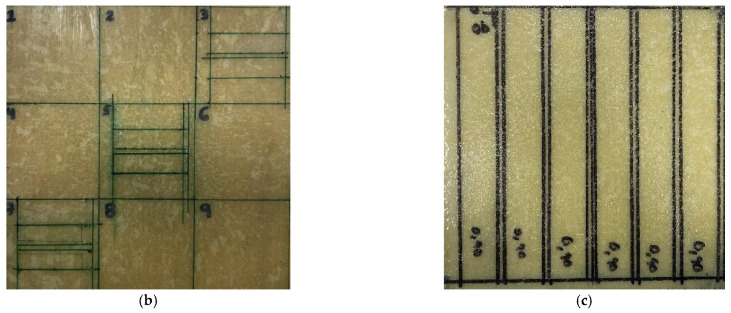
Pictures corresponding to the GF laminates manufacturing: (**a**) front view of closed hot plates with temperature and pressure displays, (**b**) GF laminate fabricated at 200 °F (93 °C) during 1 h, (**c**) GF laminate fabricated at 300 °F (149 °C) during 2 h.

The GF laminates prepared using distinct manufacturing parameters did not show relevant physical properties like changes in color or uniformity (Figure 4).

**Figure 5:**
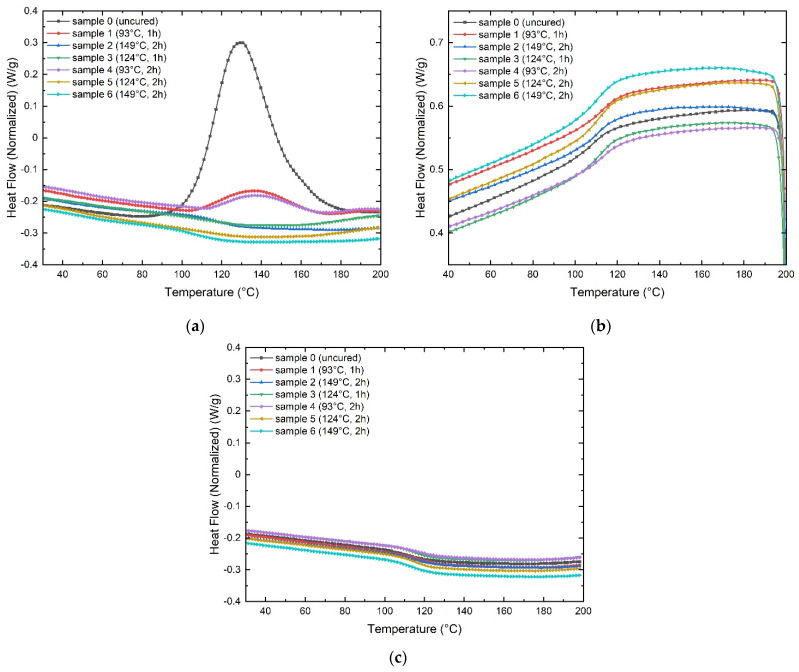
DSC curves corresponding to the samples 0 to 6: (**a**) first heating scan, (**b**) cooling step, (**c**) second heating scan.

The curing process of thermosetting resin is complex, involves a series of chemical reactions, and finally transforms the low molecular weight monomer into the macromolecular crosslinked network [18]. Figure 5 shows the DSC curves corresponding to the samples 0 to 6 (Table 1). The sample labeled as 0 corresponds to the uncured GF. The samples 1 and 4, 2 and 6, and 3 and 5 have similar time and temperature parameters.

It can be seen that sample 0 displays a significant peak from 80 °C to 190 °C. The peak is associated with the uncured GF curing process, and it is the reference for the rest of the samples. The samples 1–4 show an evident shoulder signal, which indicates the temperature of 200 °F (93 °C) is not sufficient to complete curing. The samples 2–6 reveal a slope change from approximately 115 and 100 °C, respectively. The samples 2–6 and 3–5 do not reveal any peak or shoulder signals, representing the manufacturing parameters used for the fabrication of these GF laminates that are effective for the curing process. The cooling scan does not reveal relevant changes in the curing process of GF laminates. The second heating scan reveals that the GF laminate samples are entirely cured. Lvtao Zhu et al. [19] presented similar results in a recent work.

**Figure 6:**
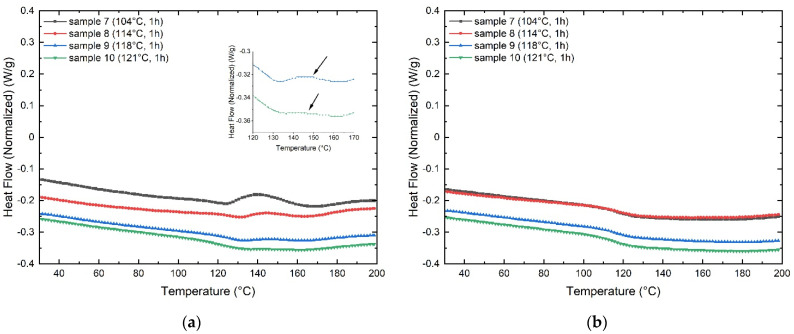
DSC curves corresponding to the samples 7 to 10: (**a**) first heating scan, (**b**) second heating scan.

The previous observations show that time is not relevant for the curing process, the reason sample 4 can be discarded. The downward slope of the sample curves 2 and 6, manufactured at 300 °F (149 °C), represents the fact that these GF laminates were cured with excessive energy. The slope change was not so pronounced in samples 3 and 5. Therefore, the next step is to find the optimal cure temperature set at 200 psi pressure and 1 h for cure time, but reducing the temperature within a 6 °F range (250, 244 and 238 °F) (121, 118 and 114 °C), and the half between 238 °F (114 °C) and 200 °F (93 °C) because it was observed that curing temperature of 200 °F (93 °C) represents uncured GF. Figure 6 shows the DSC curves corresponding to the samples 7 to 10 (Table 1).

The samples 7 and 8 display an evident shoulder signal, which is much less evident in sample 9. Sample 10 shows imperceptible features of uncured GF laminate. However, an inset graph allows visualizing the presence of a very slight shoulder pointed by an arrow. The second heating scan does not present significant variations, representing that the GF laminate samples are entirely cured. Therefore, the optimal manufacturing process for the GF laminates results in 200 psi of pressure at 256 °F (124 °C) for 1 h.

**Figure 7:**
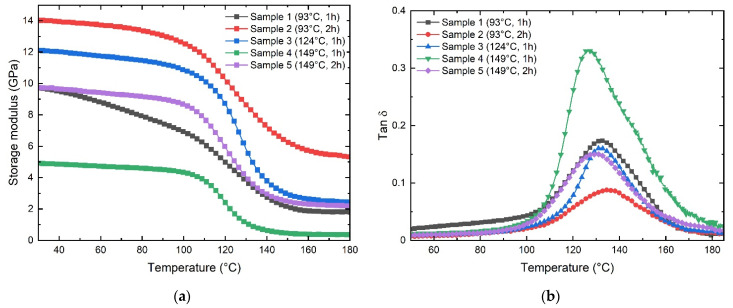
DMA curves corresponding to the samples 1 to 5: (**a**) storage modulus, (**b**) tan δ.

The viscoelastic properties like storage modulus and the loss factor, or tan δ, were obtained as a function of the temperature from the glassy state to the rubbery plateau. The DMA curves show the viscoelastic response of GF laminates fabricated with distinct manufacturing parameters, as presented in Figure 7.

From Figure 5a, it is possible to appreciate that the manufacturing parameters strongly influence GF laminates’ mechanical properties. The storage modulus varies depending on the manufacturing parameters. When comparing samples 1–2 and 4–5, it is observed that the storage modulus increases markedly with the cure time by 44% and 98%, respectively. When comparing samples 1–4 and 2–5, it is appreciated that the storage modulus decreases notoriously when the GF laminates are cured at elevated temperatures by 48% and 67%, respectively. Despite similar modulus values being obtained in samples 1–5, the behavior of the storage modulus of sample 1 reveals that the stiffness decreases markedly with increasing temperature. In fact, the value of the starting temperature (Tonset) of the transition from the glassy phase to the rubber region could not be determined. This behavior is attributed to incomplete curing. Table 2 presents the values obtained from the storage modulus, the starting temperature (Tonset), and the T_g_ obtained from the Tan δ plot (Figure 7b).

The glass transition temperature (T_g_) of laminate composites is commonly obtained from the Tan δ peak in the glass transition region, where the material changes from rigid to more elastic. It is associated with the intermolecular movement of polymer chains [20,21]. Figure 7b shows the effect of the manufacturing parameters on the T_g_ of GF laminates. Sample 4 shows the lowest T_g_ value located at 126.7 °C, and sample 2 the highest one at 134.7 °C. The rest of the samples do not present significant variations.

Compression tests for HC were performed under displacement control at a crosshead speed of 0.5 mm/min. The HC followed the ASTM C365/C365M–16 procedures to determine compressive strength and modulus of sandwich cores.

**Figure 8:**
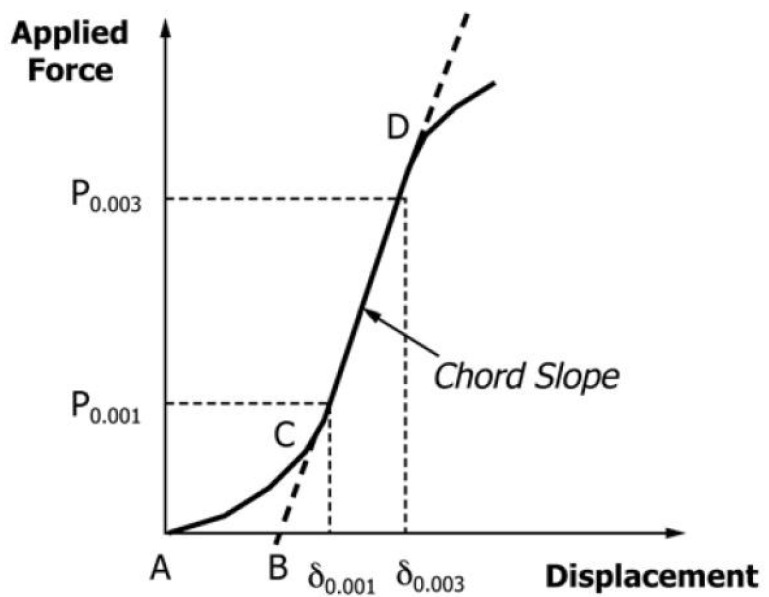
Force vs. displacement with Hookean region of core material.

The region of AC in Figure 8 is caused by the take-up of slack and alignment or seating of the specimen and does not represent the material properties. The zero points on the displacement axis have to be corrected. The linear region CD has to be extended and constructed through the zero-force axis to receive point B, the corrected zero displacement point (δ = 0.0000) from which to measure all displacements.

For the 2% deflection stress, the load-displacement curve is altered by removing the toe region in the first segment. A linear curve achieves this between the 25% and 50% of maximal load points [22]. All data before this new zero point are removed and divided by the honeycomb thickness. Afterward, the nearest value to 0.02 is searched, and displacement and load at this point are extracted from the data. As the standard demands for the compressive modulus, the displacement values should be such that δ/t is closest to 0.003 and 0.001.

**Figure 9:**
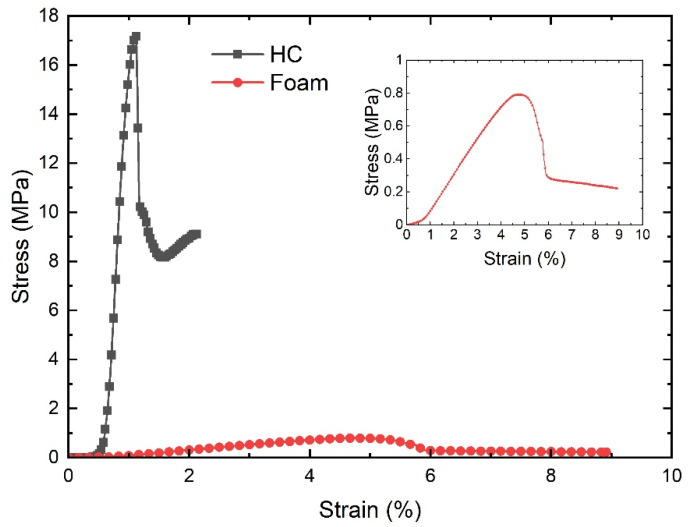
Stress vs. strain curves corresponding to the honeycomb core (HC) and foam tested under compression.

Following the procedure stated in the ASTM D1621.1873 [23], foam’s compressive modulus and apparent modulus are calculated. Additionally, the ultimate compressive strength is derived from the test data. By continuing the linear region of the load-displacement curve with load values at 25% and 50% of maximal load, the toe region in the first segment is compensated by obtaining the new zero point where the linear curve intersects with the *x*-axis. With this new zero point, the compressive strength is calculated to be like that outlined in the standard. As this compressive strength accounts for the maximal load when the foam cells are not fully compressed, the ultimate compressive strength at the highest load has been calculated. At this point, the cells are fully compressed, and there are no air bubbles within the material. The compression is conducted until a yield point or until the specimen has been compressed 13% of original thickness is reached. Figure 9 shows the stress vs. strain curves corresponding to the HC and foam cores.

As expected, the HC structure contains a rigid element configuration that promotes remarkable strength under the compression test. The foam results in meager strength due to the intrinsic properties of the material and the multicellular configuration that collapses with a low level of stress. However, the foam shows large deformations and some remaining strength (crushed strength) after cells are compressed. The previous phenomenon was encountered by Rene Roy et al. [24]

The compressive modulus of the HC was 99.3 MPa, while the modulus of the foam was 11.8 MPa. The HC can support a load greater than 13% of its compression compared to the foam. However, foam shows greater elasticity due to the plastic structure with the air cavities that form it [25].

In CATIA V5^®^ the shape of a basic business or first-class seat panel is modeled. The model’s thickness is composed of two plies of prepreg on each facesheet with core in between, following 13.3 mm total thickness. The panel is imported into ANSYS^®^ as a .stp file to use the shell structure.

In order to compare the new material approach of GF face sheets and foam core, a baseline sandwich material is used and computed with the same fixtures and load. The baseline sandwich material consists of material in ANSYS Engineering Data Sources where Epoxy E-Glass Wet for the facesheets and an essential honeycomb core are selected. For the study case of glass fiber with foam core, the experimental data are inserted in Engineering Data Sources.

**Figure 10:**
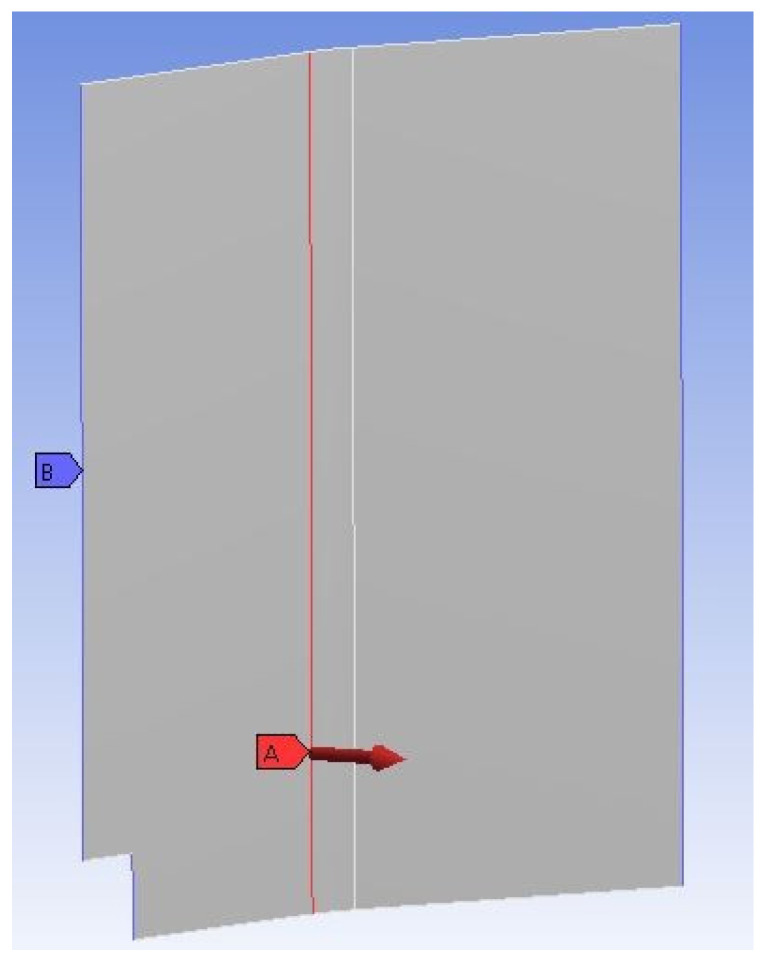
Side panel with fixed support and applied load.

The model in ACP (Pre) will mesh with a mesh size of 10 mm, and subsequently, in the setup, the material data with fabrics and stack-ups are defined. After the ACP (Pre) process is completed, this building block is linked to Static Structural and the ACP post-processor. In the Static Structural setup a force with the magnitude half of the max. sandwich load resulting in 400 N is introduced to one edge, and the panel is fixed with fixed supports at the three outer edges, as seen in Figure 10.

With Static Structural it is possible to compute a ply-wise solution of composites. Here the total deformation, equivalent elastic strain, and equivalent stress were computed and displayed and we describe the stress results in more detail below.

**Figure 11:**
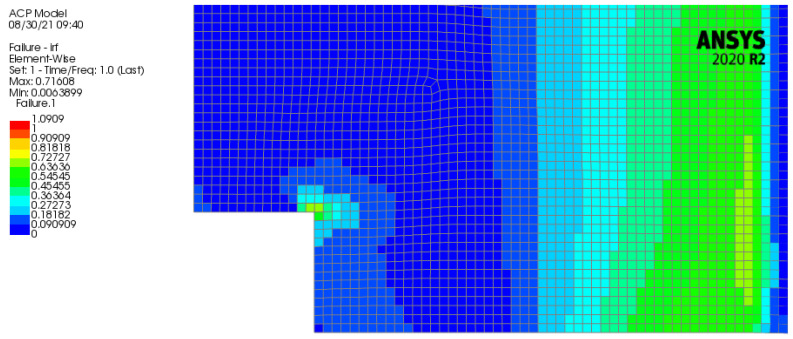
Applicable failure criterion and related ply in brackets in the bottom left corner of the baseline panel.

In ACP (Post), a more suitable analysis for composites is possible, so this post-processor was used to visualize some detailed results. Total deformation, stress, and strain, as well as ply-wise stress and strain, were computed. With the failure criteria tool in ANSYS some reinforced ply criteria and sandwich criteria can be selected like Tsai-Wu, Hashin, Puck, Max. Strain, Max. Stress or Face Sheet Wrinkling, Core Failure, and Shear Crimping. These failure criteria give some additional information on which criteria are applicable for which ply, as seen in Figure 11.

**Figure 12:**
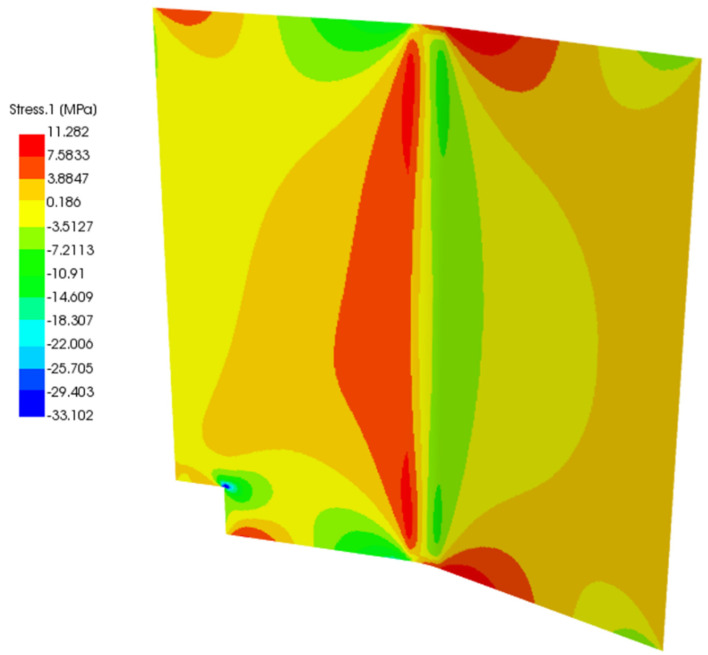
Total stress of the baseline panel in ACP.

**Figure 13:**
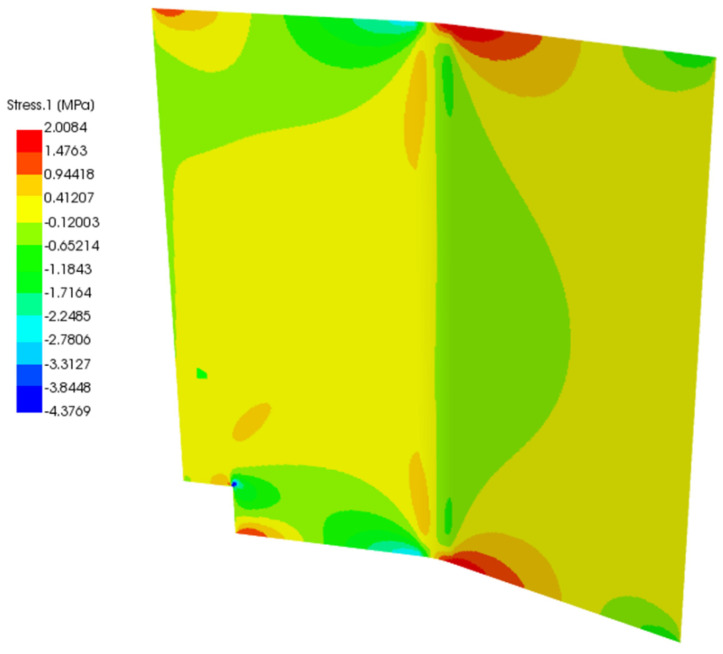
Total stress of the study case panel in ACP.

In Figure 12 and Figure 13, the baseline and the second study case solution of total stress are displayed. As the load was applied in the middle of the panel, it shows mirrored displacement, stress, and strain. For the total stress in the baseline panel, a maximum value of 11.282 MPa and a maximum negative value of −33.102 MPa, which means compression stress, are calculated. Stress peaks occur because the panel is bent, and there is material missing in the bottom left corner. The undulation of the panel with the additional force in the middle of the panel leads to the high-stress values in the center. The high stresses derive from an edge effect on the upper and lower sides for baseline and study case panel.

The study case panel with the glass fiber prepreg and foam core exhibits a much lower stress magnitude than the baseline panel. The center does not show any additional maximum stresses and has a maximum positive stress value of 2.0084 MPa and a maximum negative stress value of −4.3769 MPa.

What has to be considered is the lower-left corner, where some material is missing in the panel. Here stress peaks occur, which are analyzed with a composite failure criteria in ACP(Post). These compression stress peaks will result in the zone of the panel shown in Figure 13. The green color of the section in the corner refers to a value between 0.6–0.725. For the Tsai-Wu criterion, which will be looked at, the panel will fail if the magnitude of the factor reaches the red area (values up to 1). In the baseline panel larger values of the failure criterion are exhibited with respect to the study case panel with a maximum of 0.25 in the zone analyzed in Figure 11.

## 4. Conclusions

Ever more research is being undertaken on composite material processing geared towards the aviation industry. Composites are used in aircraft interiors due to their advanced features and their ability to reduce the aircraft’s overall weight, optimizing fuel consumption.

This work evaluated the different manufacturing parameters for fiberglass prepreg curing. The technical sheets of the materials provide a starting point but should not be considered for the development of industrial parts. The detailed analysis allows finding the optimal manufacturing conditions that affect the mechanical performance of the compounds and the useful life of the components. The use of analytical tools such as DSC and DMA allows a complete evaluation of the curing conditions of fiberglass prepreg in the manufacture of sandwich compounds.

Polymeric foams do not provide remarkable mechanical strength compared to honeycomb structures. However, the foams showed much more significant deformation if they did break or permanently deform, allowing their use for non-structural applications.

Modeling mechanical behavior with the finite element method in ANSYS and any other modeling software requires a deep understanding of the theoretical background. Otherwise, the evaluation will not be productive. A variety of options are possible, the direction of stress or strain of which should be analyzed ply-wise or for the whole panel. The evaluation with the same load and supports was presented for the baseline and the study case (proposed material) panels. It was shown that the proposed material in the study case panel exhibits areas with lower stress concentrations with respect to the baseline, especially in the middle zone of the panel. This is also reflected when the Tsai-Wu failure criterion is applied to the panels, in which the maximum value of the failure factor is substantially smaller in the study case panel with respect to the baseline.

The panel modeled in this work is thought to be an approximation of a seat panel as it was not possible to use original dimensions. Also, the student version is limited to a defined node number and does not use the whole software capabilities. Further research should be undertaken for specific data by evaluating a unique panel with expected loads for different use cases.

The results allow the continuation of research activities in the development of panels at the prototype level. A second article will provide relevant information on the manufacturing processes and alternative materials to manufacture non-structural panels for aircraft cabin interiors, based on the results obtained in this article.

## Figures and Tables

**Table 1:** Manufacturing parameters of glass fiber (GF) laminates.

GF Laminate	Pressure (psi)	Compression Temperature (°F/°C)	Pressure Time (h)
0	-	-	-
1 *	20	200/93	1
2	20	300/149	1
3	20	256/124	1
4	20	200/93	2
5	20	256/124	2
6	20	300/149	2
7	20	219/104	1
8	20	238/114	1
9	20	244/118	1
10	20	250/121	1

* Manufacturing parameters recommended by the supplier.

**Table 2:** DMA parameters obtained.

Sample	Temperature (°F/°C)	Time (h)	Storage Modulus (GPa)	Tonset (°C)	T_g_ (°C)
1	200/93	1	9.72	---	132.3
2	200/93	2	14	98.5	134.7
3	256/124	1	12.1	107.4	132.6
4	300/149	1	4.91	106.4	126.7
5	300/149	2	9.74	102.4	129.8

## Data Availability

This study did not report any data.
